# Histone Deacetylase 3 and 4 Complex Stimulates the Transcriptional Activity of the Mineralocorticoid Receptor

**DOI:** 10.1371/journal.pone.0136801

**Published:** 2015-08-25

**Authors:** Hae-Ahm Lee, Min-Ji Song, Young-Mi Seok, Seol-Hee Kang, Sang-Yeob Kim, Inkyeom Kim

**Affiliations:** 1 Department of Pharmacology, School of Medicine, Kyungpook National University, Daegu, 41944, Republic of Korea; 2 Cardiovascular Research Institute, School of Medicine, Kyungpook National University, Daegu, 41944, Republic of Korea; 3 Cell and Matrix Research Institute, School of Medicine, Kyungpook National University, Daegu, 41944, Republic of Korea; 4 Korea Promotion Institute for Traditional Medicine Industry, Gyeongsan, Gyeongbuk 712–260, Republic of Korea; 5 Asan Institute for Life Sciences, Asan Medical Center, Seoul 138–736, Republic of Korea; 6 Department of Medicine, University of Ulsan, College of Medicine, Seoul 138–736, Republic of Korea; Saint Louis University School of Medicine, UNITED STATES

## Abstract

Histone deacetylases (HDACs) act as corepressors in gene transcription by altering the acetylation of histones, resulting in epigenetic gene silencing. We previously reported that HDAC3 acts as a coactivator of the mineralocorticoid receptor (MR). Although HDAC3 forms complexes with class II HDACs, their potential role in the transcriptional activity of MR is unclear. We hypothesized that HDAC4 of the class II family stimulates the transcriptional activity of MR. The expression of MR target genes was measured by quantitative real-time PCR. MR and RNA polymerase II recruitment to promoters of MR target genes was analyzed by chromatin immunoprecipitation. The association of MR with HDACs was investigated by co-immunoprecipitation. MR acetylation was determined with an anti-acetyl-lysine antibody after immunoprecipitation with an anti-MR antibody. Among the class II HDACs, HDAC4 interacted with both MR and HDAC3 after aldosterone stimulation. The nuclear translocation of HDAC4 was mediated by protein kinase A (PKA) and protein phosphatases (PP). The transcriptional activity of MR was significantly decreased by inhibitors of PKA (H89), PP1/2 (calyculin A), class I HDACs (MS-275), but not class II HDACs (MC1568). MR acetylation was increased by H89, calyculin A, and MS-275, but not by MC1568. Interaction between MR and HDAC3 was significantly decreased by H89, calyculin A, and HDAC4 siRNA. A non-genomic effect of MR via PKA and PP1/2 induced nuclear translocation of HDAC4 to facilitate the interaction between MR and HDAC3. Thus, we have uncovered a crucial role for a class II HDAC in the activation of MR-dependent transcription.

## Introduction

Our previous study revealed that histone deacetylase (HDAC) facilitates transcriptional activity of mineralocorticoid receptor (MR) [1]. HDACs are important enzymes in epigenetic gene silencing, acting as corepressors of transcription by deacetylating the ε-amino group of histone lysine residues. Thus far, over a dozen HDACs have been discovered and grouped into distinct subfamilies according to their amino acid sequence similarities and structural features [2]. Class I HDACs (HDAC1, 2, 3, and 8) are predominately nuclear whereas class II HDACs (HDAC4, 5, 6, 7, 9, and 10) are expressed in a cell-type specific manner and shuttle between the nucleus and the cytoplasm [3]. Class II HDACs are further divided into class IIa (HDAC4, 5, 7, and 9) and class IIb (HDAC6 and 10). Several studies have revealed that class IIa HDACs are catalytically inactive because of critical amino acid substitutions within their active sites [4–7]. Although class IIa HDACs show limited enzymatic activity, they function as important transcriptional repressors by recruiting corepressors to promoters [4, 7].

The subcellular localization of class IIa HDACs is controlled by phosphorylation of specific serine residues in the N-terminal region by several protein kinases including calcium/calmodulin-dependent protein kinase (CaMK), salt-inducible kinase (SIK), and protein kinase D [8–10]. Phosphorylation of the HDACs by these kinases promotes their interaction with 14-3-3 proteins, resulting in cytoplasmic retention and activation of their target genes [11]. Dephosphorylation of class IIa HDACs by protein phosphatases (PP) such as PP1, PP2, and myosin phosphatase leads to their dissociation from 14-3-3 proteins, nuclear import, and recruitment of repressor proteins to target genes [7, 8]. However, phosphorylation of class IIa HDACs can also promote their nuclear translocation. Protein kinase A (PKA) not only promotes the nuclear import of class IIa HDACs by phosphorylating serine-proline motifs in HDAC4 [12], but also inhibits the activity of protein kinases including SIK1, 2, and 3 to attenuate HDAC cytoplasmic retention [13]. In addition, PKA activates PP2A which removes phosphates on conserved 14-3-3 binding sites of class IIa HDACs [14]. Therefore, PKA, PP1, and PP2 play a central role in the translocation of class IIa HDACs from the cytosol to the nucleus.

It is well established that the catalytic activity of HDAC4 does not play a role in inhibiting the transcriptional activity of myocyte enhancer factor 2 (MEF2). However, HDAC4 binds directly to MEF2 and recruits class I HDACs to form a repressive complex in the nucleus [15]. Several transcription factors such as serum responsible factor, nuclear factor of activated T-cells, runt-related transcription factor, GATA-binding proteins, and cAMP response element-binding protein are also repressed by class IIa HDACs in a catalytic activity-independent manner [16]. Therefore, non-catalytic functions of class IIa HDACs (*e*.*g*. mediators and scaffolds) have been suggested [17].

HDACs are generally described as transcriptional repressors; however, recent studies have reported that HDAC inhibitors can actually repress the expression of some genes [18, 19]. Several pieces of evidence have shown that the transcriptional activity of nuclear receptors and other transcription factors is impaired by their acetylation [17, 18, 20, 21]. This is one mechanism by which HDAC inhibitors can repress the transcription of some genes despite global hyperacetylation of histones. Our previous study also showed that HDAC inhibitors attenuate the transcriptional activity of the mineralocorticoid receptor (MR) by increasing acetylation in the MR hinge region, reducing its affinity for target gene promoters. HDAC3 is the sole enzyme responsible for the deacetylation of kidney MR [1]. However, the molecular mechanism by which class II HDACs interact with MR or HDAC3 has not been well characterized. We hypothesized that the HDAC3/HDAC4 complex stimulates the transcriptional activity of MR and we used HDAC class-specific inhibitors to eliminate HDAC activity or siRNA to deplete cells of the HDACs to determine whether class II HDACs have a role in MR activation. Moreover, we investigated the PKA and PP1/2 signaling pathway which regulates the nuclear translocation of class II HDACs.

## Materials and Methods

### Cell culture

Human embryonic kidney cells (HEK293) were cultured in Dulbecco's Modified Eagle Medium with 10% fetal bovine serum (FBS), 50 μg/ml penicillin and streptomycin at 37°C in a 5% CO_2_ humidified incubator. The normal growth media was changed with 2.5% charcoal-stripped FBS before aldosterone (Aldo, 10 nmol/L) stimulation with vehicle, H89 (10 μmol/L), Calyculin A (5 nmol/L), MS-275 (10 μmol/L), or MC1568 (10 μmol/L) pretreatment for 6 h.

### Plasmids

The pCMV-HA-MR was kindly donated by Dr. Tirard (Max-Planck-Institute). A plasmid for fluorescent resonance energy transfer (FRET)-based PKA indicator (AKAR3EV) was provided by Prof. Matsuda (Kyoto University). HDAC4-flag (Addgene plasmid 13821), HDAC5-flag (Addgene plasmid 13822), HDAC7-flag (Addgene plasmid 13824), pEGFP-wt-HDAC4 (Addgene plasmid 45636), pEGFP-3SA HDAC4 (Addgene plasmid 45637), and HDAC4-3SA-flag (Addgene plasmid 30486) vectors were purchased from Addgene.org. For transfection, 2 μg and 10 μg of plasmid were used for 6 well plates and 100 mm culture dishes respectively.

### Quantitative real-time PCR (qRT-PCR)

For gene expression study, HEK293 cells (2X10^5^) were seeded in 6 well plates. After 24 h, cells were pretreated with vehicle or inhibitors and then stimulated with Aldo for 24 h. Trizol Reagent (Invitrogen, Carlsbad, CA) was used to extract total RNA according to manufacturer’s instruction. Total RNA (2 μg) was reverse-transcribed into cDNA by using RevertAid^TM^ first strand cDNA synthesis kit (Fermentas, EU). Quantitative real-time-PCR (qRT-PCR) was performed using SYBR Green PCR master mix (TaKaRa, Japan) and ABI Prism 7500 sequence detection system (Applied Biosystems, Foster City, CA). The relative gene expression level was determined by Δcycle threshold (ΔCt) compared with its endogenous control (*Gapdh*). Primer sequences in the present study are shown in S1 Table.

### Chromatin immunoprecipitation (ChIP) assay

For ChIP assay, HEK cells (1X10^6^) were seeded in 100 mm culture dishes. The cells were transfected HA-MR. After 24 h of transfection, cells were stimulated with Aldo (10 nmol/L) for 30 min with or without pretreatment with inhibitors (MC1568, MS-275, H89 or CalA). ChIP analysis was performed by using EZ ChIP kit (Upstate Biotechnology, Lake Placid, NY) as described previously [1]. Enrichment of MR on promoters of target gene was determined by qRT-PCR. To confirm the specific enrichment of MR on HRE of MR target gene promoter, about 2 Kb upstream of gene promoters were amplified as a negative control.

### Immunoprecipitation and Western blot

HEK293 cells (2X10^5^ in 6 well plates) were transfected with MR-HA and HDAC-Flag plasmids by using Superfect reagents (Qiagen, Hilden, Germany) according to manufacturer’s recommendation. After 48 h, cells were stimulated with Aldo with or without pretreatment with inhibitors for 6h. Cell were lysed by a lysis buffer (20 mmol/L Tris, 150 mmol/L NaCl, 1% NP-40, 0.5% sodium deoxycholate, 0.1% sodium dodecyl sulfate, and 1x proteinase inhibitor cocktail). The cell lysates (100 μg) were precleared with protein G agarose at 4°C for 2 h. After centrifugation (3000 g for 2 min), supernatants were incubated with 1μg of hemagglutinin (HA) antibody (Cell Signaling Technology, Beverly, MA) at 4°C for overnight. The immunocomplexes were washed with the lysis buffer (three times), and boiled after adding 2X protein sample buffer. To detect Flag tagged HDAC, Western blotting was used as described previously [1].

### FRET imaging

LLC-PK1 cells were cultured on a collagen-coated 35-mm glass-base dish and were transfected with AKAR3EV plasmid using Lipofectamin transfection reagent, according to the manufacturer’s instruction. To analyze PKA activation, FRET was detected as described previously [1]. In brief, FRET images were obtained by a Nikon Ti-E inverted microscope equipped with PFS, CoolSNAP HQ camera (Roper Scientific, Trenton, NJ), excitation and emission filter wheels. Images were acquired by using the 4×4 binning mode and 200-ms exposure time. All systems were controlled by MetaMorph software. For dual-emission ratio imaging of the intramolecular FRET probe, we obtained images for cyan fluorescent protein (CFP) and FRET. After background subtraction, pseudo-color images of FRET/CFP were created using eight colors from red to blue to represent the FRET/CFP ratio via the intensity modulated display (IMD) mode by using MetaMorph software. Filters used for the dual-emission ratio imaging were purchased from Semrock: an FF01-438/24-25 excitation filter, an FF458-Di02-25×36 dichroic mirror, and two emission filters, FF01-483/32-25 for CFP and FF01-542/27-25 for FRET.

### Statistics

Results are expressed as mean±SEM. Kruskal–Wallis test and 1-way ANOVA followed by post hoc Tukey’s comparison test were used. Differences were considered as significant at *P*<0.05. The Student *t*-test was applied for analysis of significant differences between the 2 groups. The procedures were performed using SPSS software (release 19.0, SPSS Inc, Chicago, IL).

## Results

### Aldosterone-induced MR interaction with HDAC3 and HDAC4

**Fig 1:**
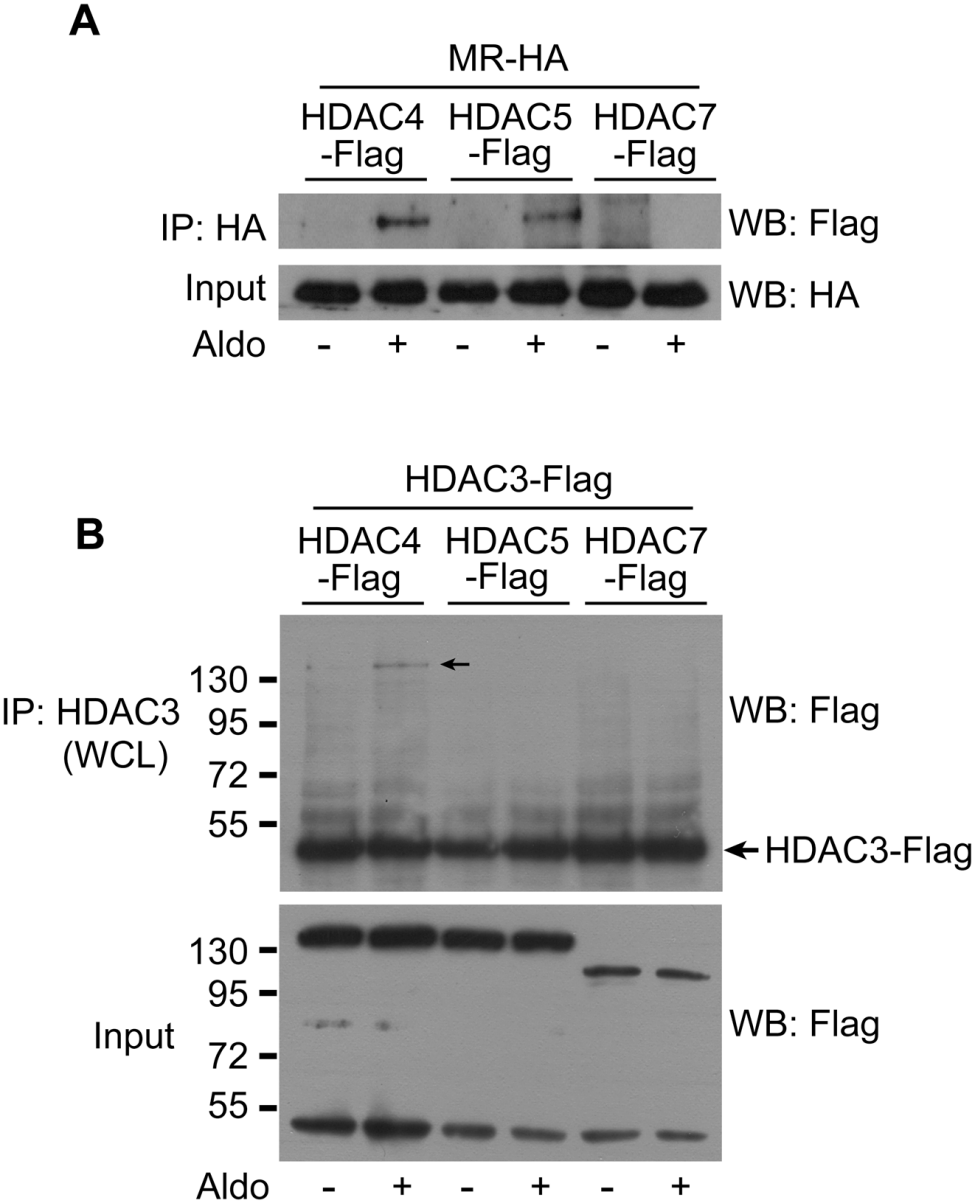
HDAC4 interacts with both the MR and HDAC3. A, HEK293 cells were transfected with MR-HA and either HDAC4-Flag, HDAC5-Flag, or HDAC7-Flag plasmids. After 48 h, the cells were treated with Aldo (10 nM) for 30 min. Whole-cell lysates (WCLs) were prepared with non-denaturing lysis buffer. The MR was precipitated with an anti-HA antibody and MR-interacting HDACs were detected by western blotting with an anti-Flag antibody. HDAC4 and HDAC5 interacted with MR after Aldo treatment. B, HEK293 cells were transfected with HDAC3-Flag and HDAC4-Flag, HDAC5-Flag, or HDAC7-Flag plasmids. HDAC3 was immunoprecipitated with an antibody and HDAC3-interacting class II HDACs were detected by western blotting. HDAC4 interacted with HDAC3 after Aldo treatment. Aldo, aldosterone; HA, hemagglutinin; IP, immunoprecipitation; WB, western blotting.

We previously reported that HDAC3 binds to MR. HDAC3 deacetylates MR, which increases its transcriptional activity [1]. Similar to our results, Mihaylova et al. showed that HDAC3 deacetylates the forkhead box O (FOXO) transcription factor, the interaction of which is mediated by class IIa HDACs [17]. To identify the role of class II HDACs in the interaction between MR and HDAC3, HA-tagged MR and Flag-tagged HDAC4, 5, and 7 were co-transfected into HEK293 cells and co-immunoprecipitation (co-IP) was performed to investigate the interaction between MR and class II HDACs. HDAC4 and HDAC5 interacted with MR stimulate by Aldosterone (Aldo) (Fig 1A). To identify a class II HDAC that simultaneously binds to the MR and HDAC3, HDAC3 and each of the class II HDACs were co-expressed in HEK293 cells. Treatment with Aldo promoted an interaction between HDAC3 and HDAC4 but not with the other class II HDACs (Fig 1B). Together, these results indicate that HDAC4 interacts with MR regulators and may also play an important role in MR function.

### Transcriptional activity of MR is repressed by inhibiting class I but not class II HDACs and also is influenced by HDAC4 levels

**Fig 2:**
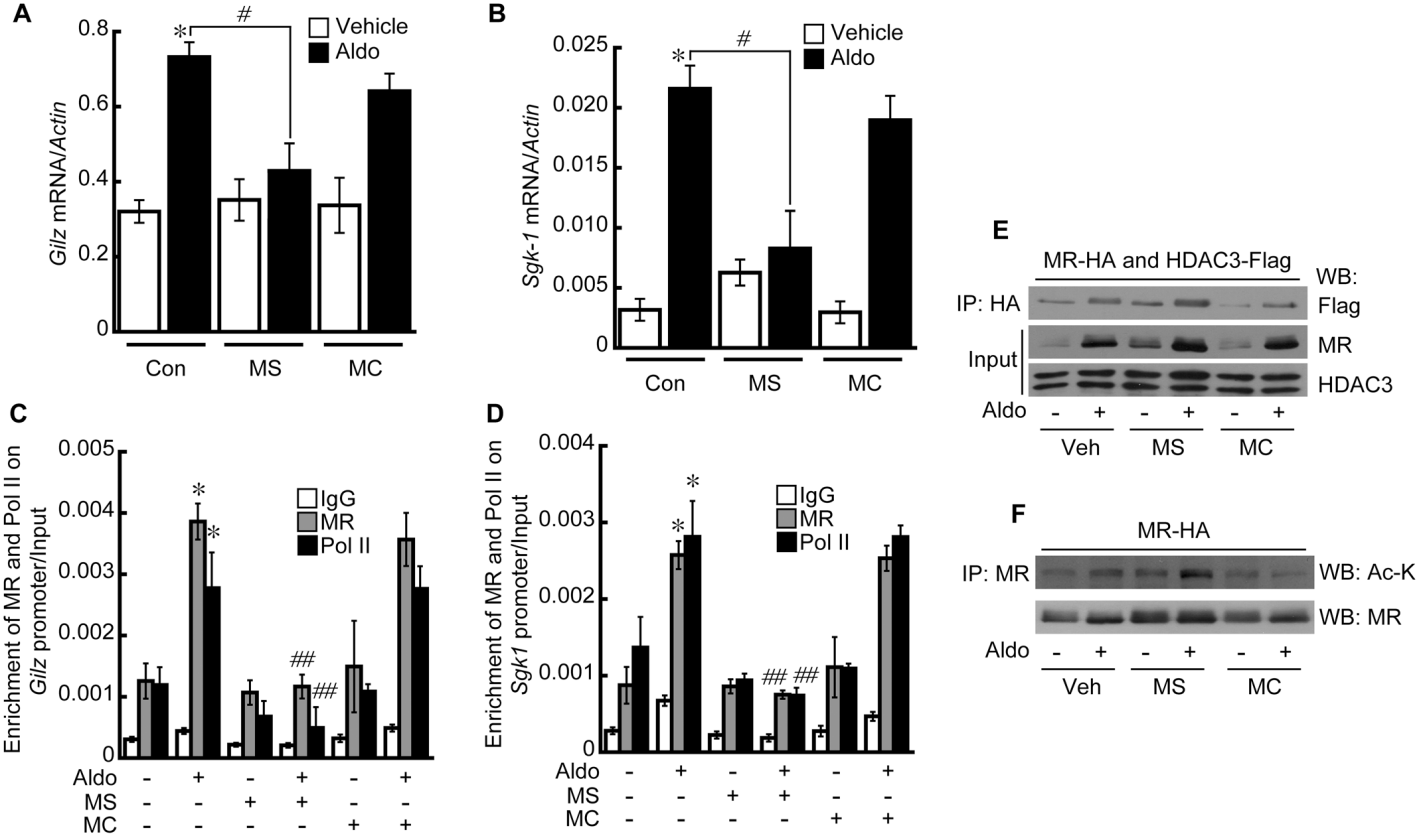
Transcriptional activity of the MR is attenuated by MS-275 but not MC1568. HEK293 cells were stimulated with Aldo (10 nM) for 24 h after pretreatment with MS-275 (10 μM), a class I HDAC inhibitor, MC1568 (10 μM), a class II HDAC inhibitor, or vehicle for 6 h. Treatment with Aldo resulted in increased expression of *GILZ* (A) and *SGK-1* (B), which was decreased by MS-275 but not by MC1568. Graph shows the means ± SE of three independent experiments (*p < 0.05 vs. vehicle, ^#^ *p* < 0.05 Aldo vs. MS+Aldo). Recruitment of MR and Pol II to *GILZ* (C) and *SGK-1* (D) was increased by Aldo, which was inhibited by MS-275 but not by MC1568. Graph shows the means ± SE of three independent experiments (*p < 0.05 vs. vehicle, ^##^ *p* < 0.01 Aldo vs. MS+Aldo). E, The interaction between MR and HDAC3 was increased by Aldo treatment, which was not affected by MS-275 and MC1568 in the nucleus of HEK293 cells. F, Acetylation of MR was increased by MS-275 but not by MC1568 when HEK293 cells were stimulated by Aldo.

**Fig 3:**
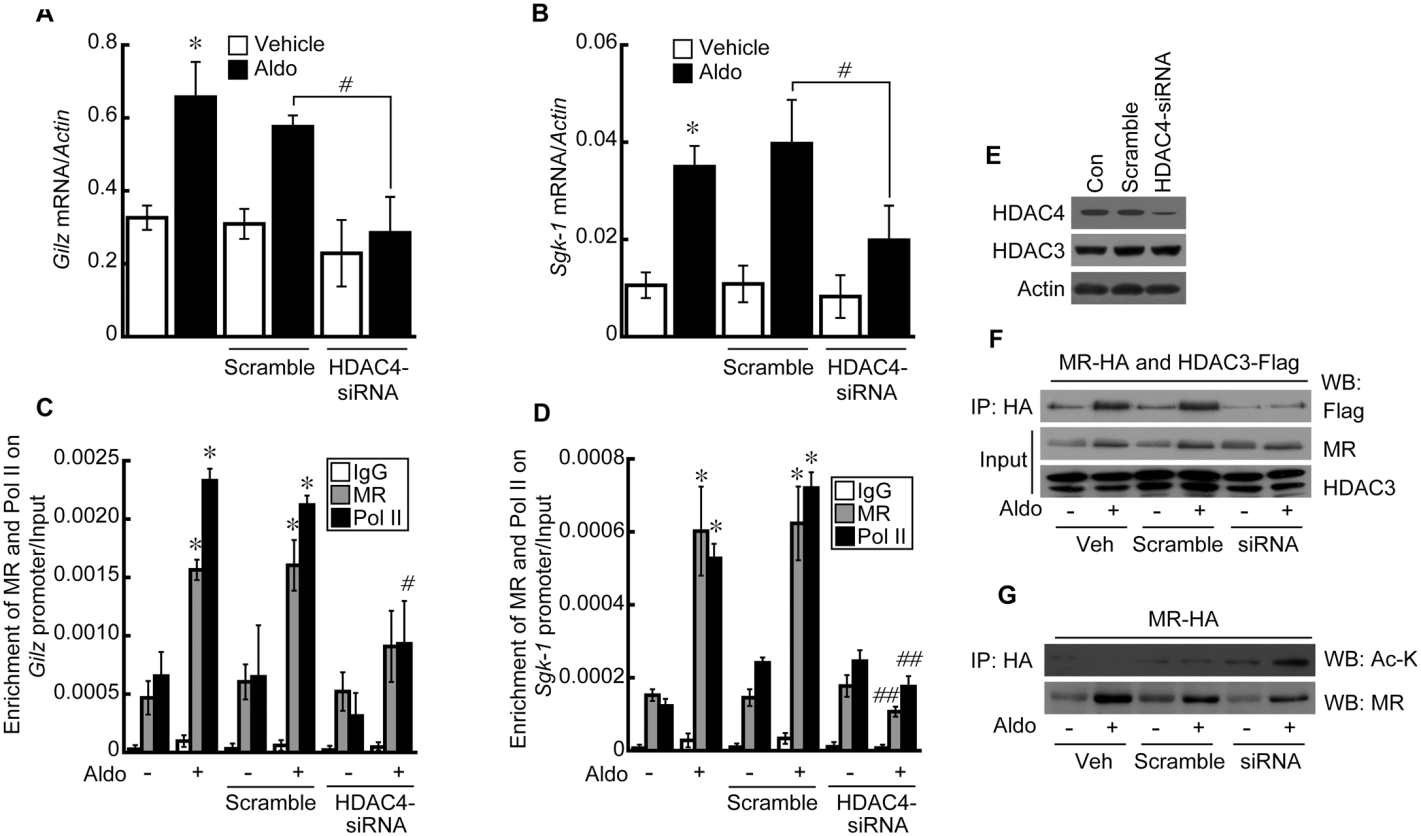
HDAC4 acts as a scaffold between the MR and HDAC3. Treatment with Aldo resulted in increased expression of *GILZ* (A) and *SGK-1* (B), which was decreased by knockdown of HDAC4. Graph shows the means ± SE of three independent experiments (*p < 0.05 vs. vehicle, ^#^ *p*<0.05 Scramble vs. siRNA). HDAC4 knockdown decreased the Aldo-induced recruitment of MR and Pol II to *GILZ* (C) and *SGK-1* (D) promoters. Schematic diagrams show the locations of HRE and PCR amplification after chromatin immunoprecipitation (ChIP) in *GILZ* (C) and *SGK-1* (D) promoters. Graph shows the means ± SE of three independent experiments (*p < 0.05 vs. vehicle, ^#^ *p*<0.05 Scramble vs. siRNA). E, HDAC4 protein was significantly decreased after 48 h of siRNA transfection. F, The interaction between MR and HDAC3 induced by Aldo was inhibited by knockdown of HDAC4. G, Acetylation of MR was increased by HDAC4 knockdown when HEK293 cells were stimulated by Aldo.

The effect of MS-275, a class I HDAC inhibitor (HDAC1, 2, and 3), and MC1568, a class II HDAC inhibitor (HDAC4 and 6), on MR target gene expression was investigated by qRT-PCR. Aldo treatment increased the expression of Glucocorticoid-induced leucine zipper (*GILZ)* by approximately 209% which was about 65% attenuated by MS-275 but not by MC1568 (Fig 2A). Expression of serum/glucocorticoid-induced protein kinase-1 (*SGK-1)* showed a similar pattern to that of *GILZ* in the presence of MS-275 and MC1568 (Fig 2B). Recruitment of MR and Pol II to the *GILZ* and *SGK-1* promoters was analyzed by chromatin immunoprecipitation (ChIP). The results of the ChIP assays showed that treatment with Aldo enriched MR (~306%) and Pol II (~232%) on the HRE sequence of *Gilz*, which was decreased by MS-275 (MR: ~70%; PolII: ~83%) but not by MC1568 (Fig 2C). Similarly, the enrichment of MR and Pol II on the *SGK-1* promoter was significantly induced by Aldo (MR: ~294%; PolII: ~206%), which was inhibited by MS-275 (MR: ~71%; PolII: ~74%) but not by MC1568 (Fig 2D). The interaction between MR and HDAC3 in the nucleus increased when the cells were treated with Aldo, which was unaffected by MS-275 or MC1568 (Fig 2E). To check if the enrichments were specific to the promoter regions examined, we analyzed the enrichment of MR and PolII at a 2 kb fragment upstream of each HRE. As expected, Aldo treatment did not enrich the proteins at the upstream genomic region (S1 Fig). We also analyzed MR acetylation, which reduces the DNA binding affinity of MR. HDAC3 inhibition by MS-275 significantly increased MR acetylation, whereas HDAC4 inhibition by MC1568 showed little effect on the acetylation status of MR (Fig 2F). Therefore, we speculated that HDAC4 has a role other than MR deacetylation in regulating MR activity. To address this hypothesis, we knocked down HDAC4 with siRNA and then analyzed the transcriptional activity of MR. Depletion of HDAC4 resulted in an approximate 77% and 73% decreased Aldo-induced expression of *GILZ* (Fig 3A) and *SGK-1* (Fig 3B), respectively. Recruitment of MR and Pol II to the *GILZ* (Fig 3C) and *SGK-1* (Fig 3D) promoters was significantly attenuated by knockdown of HDAC4 (*GILZ* (MR: 42%; PolII: 60%)) (*SGK-1* (MR: 83%; PolII: 67%). As expected, HDAC4 protein levels significantly decreased in HEK293 cells transfected with siRNA targeting HDAC4 but HDAC3 protein levels remained unchanged (Fig 3E). Interestingly, HDAC4 knockdown decreased the interaction between MR and HDAC3 in the nucleus of Aldo-treated HEK293 cells (Fig 3F). HDAC4 knockdown showed little effect on MR translocation to the nucleus (Fig 3F), whereas it increased Aldo-stimulated MR acetylation in the nucleus (Fig 3G). Together, these data reveal that HDAC4 has a critical role in MR transcriptional activation and increases the interaction between MR and HDAC3.

### PKA activation and HDAC4 nuclear localization is influenced by Aldo treatment

**Fig 4:**
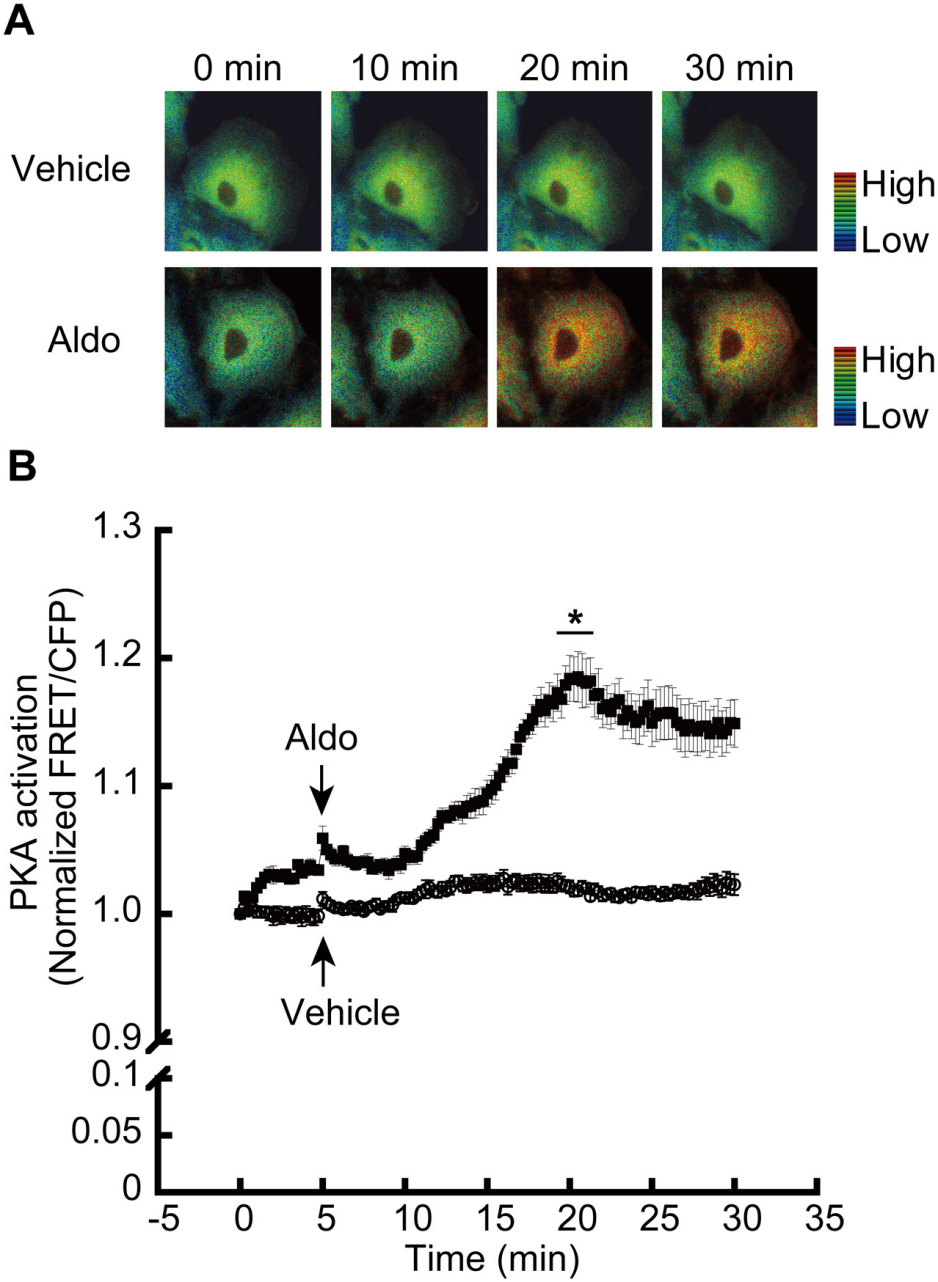
Aldo induces protein kinase A (PKA) activation. A, LLC-RK1 cells were transfected with a FRET-based indicator (AKAR3EV) construct. After 48 h of transfection, cells were adjusted with charcoal stripped fetal bovine serum-containing media (steroid hormone free). Aldo or vehicle was applied and FRET signals were captured by fluorescence microscopy. B, The FRET/CFP ratio of each cell was normalized by the averaged FRET/CFP value before stimulation. Dot graph shows the means ± SE of three independent experiments from at least 10 cells per experiment vs. time (*p*<0.05 vs vehicle).

**Fig 5:**
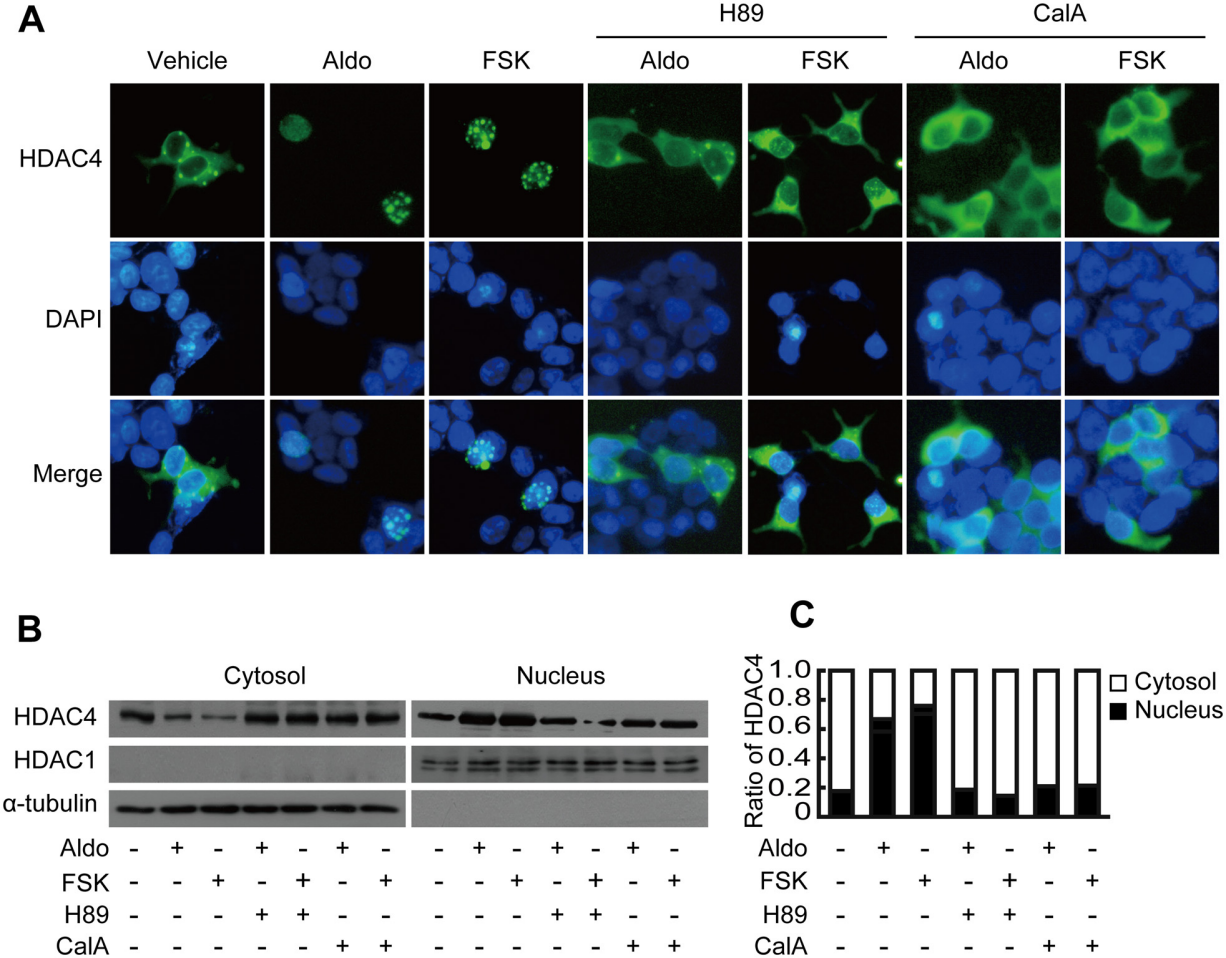
Protein kinase A (PKA) and protein phosphatase (PP) 1 and 2 are involved in the translocation of HDAC4. A, HEK293 cells were transfected with HDAC4-EGFP vectors. After 48 h of transfection, cells were treated with forskolin (FSK, 10 μM) or aldosterone (Aldo, 10 nM) for 30 min with or without pretreatment with H89 (10 μM) or calyculin A (CalA, 5 nM) for 6 h. FSK and Aldo induced the translocation of HDAC4 from the cytosol to the nucleus. Inhibition of PKA and PP1 and 2 attenuated the translocation of HDAC4 induced by FSK or Aldo. HEK293 cells were fractionated after the indicated treatment. HDAC4 was detected by western blotting (B) and quantified by densitometry (C) from cytosolic and nuclear fractions. Consistent with the fluorescence images, translocation of HDAC4 to the nucleus was increased by FSK and Aldo, which was inhibited by H89 and calyculin A.

PKA induces the nuclear accumulation of HDAC4 [22, 23]. We investigated whether Aldo treatment induces PKA activation and the nuclear accumulation of HDAC4. To investigate the Aldo-induced activation of PKA, LLC-PK1 cells were transfected with a FRET-based PKA indicator (AKAR3EV) construct. The transfected cells were treated with Aldo for thirty minutes, and the spatiotemporal activation of PKA was analyzed by FRET imaging (Fig 4A). Time courses of the FRET changes are shown in Fig 4B. Constitutively expressed cyan fluorescent protein (CFP) was used as an internal control for comparisons of FRET signals. Indeed, Aldo treatment activated PKA which showed maximal activity after fifteen minutes of Aldo treatment. The FRET/CFP ratio was unaltered in cells treated with the vehicle control. To investigate whether Aldo induced the translocation of HDAC4, HEK293 cells were transfected with the pEGFP-C2-HDAC4 plasmid. Transfected cells were stimulated with Aldo or forskolin (FSK), a PKA agonist for thirty minutes, and images of GFP fluorescence were captured by fluorescence microscopy (Fig 5A). Aldo and FSK induced the nuclear translocation of HDAC4, which was inhibited by H89 and calyculin A. To quantify the amount of HDAC4 translocation, HEK293 cell extract was fractionated into cytosolic and nuclear components, and western blotting was performed using an antibody against HDAC4. Cross-contamination was monitored with a nuclear marker (HDAC1) and a cytosolic marker (alpha-tubulin). A representative blot (Fig 5B) and quantification (Fig 5C) showed that FSK and Aldo significantly induced HDAC4 nuclear accumulation, which was inhibited by pretreatment with H89 and calyculin A.

### Inhibition of PKA, PP1, and PP2 decreases the transcriptional activity of MR

**Fig 6:**
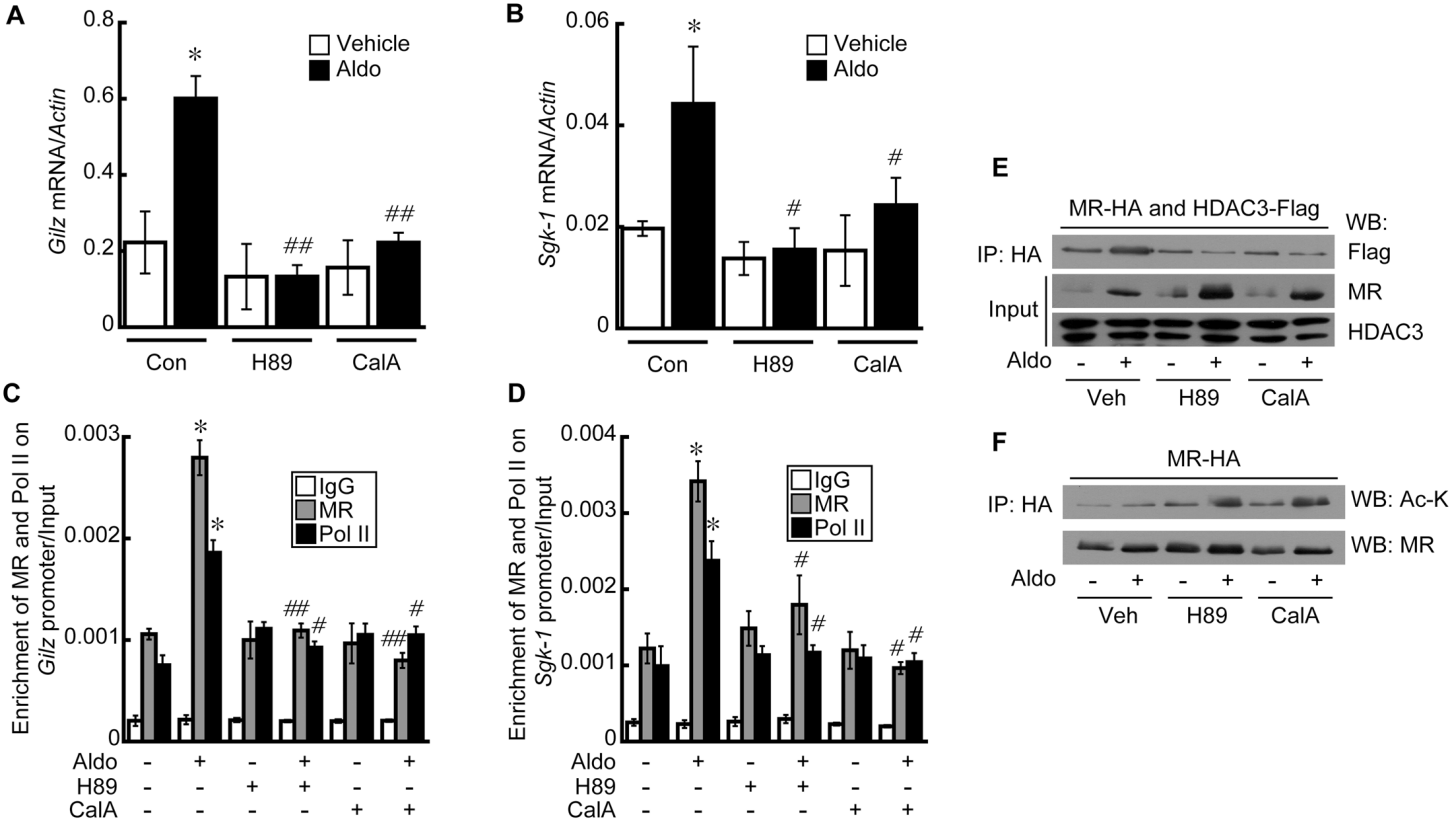
H89 and calyculin A inhibit the transcriptional activity of MR. HEK293 cells were stimulated with Aldo (10 nM) for 24 h after pretreatment with H89 (10 μM), calyculin A (5 nM), or vehicle for 6 h. Treatment with Aldo resulted in increased expression of *GILZ* (A) and *SGK-1* (B), which was decreased by pretreatment with H89 and calyculin A. Recruitment of the MR and RNA polymerase II (Pol II) to the GILZ and SGK-1 promoters was analyzed by ChIP assay. Aldo-induced recruitment of the MR and Pol II to GILZ (C) and SGK-1 (D) the promoters was inhibited by pretreatment with H89 and calyculin A. Data are the means ± SE of three independent experiments (*p < 0.05 vs. vehicle, ^#^ *p*<0.05, ^##^ *p*<0.01 Aldo vs. H89+Aldo or CalA+Aldo). HRE, hormone response element; TSS, transcription start site. E, Interaction between MR and HDAC3 was investigated by co-immunoprecipitation using an anti-MR antibody. The interaction between the MR and HDAC3 was increased by Aldo treatment, which was inhibited by H89 and calyculin A. F, Pretreatment with H89 and calyculin A increased MR acetylation when HEK293 cells were stimulated by Aldo.

**Fig 7:**
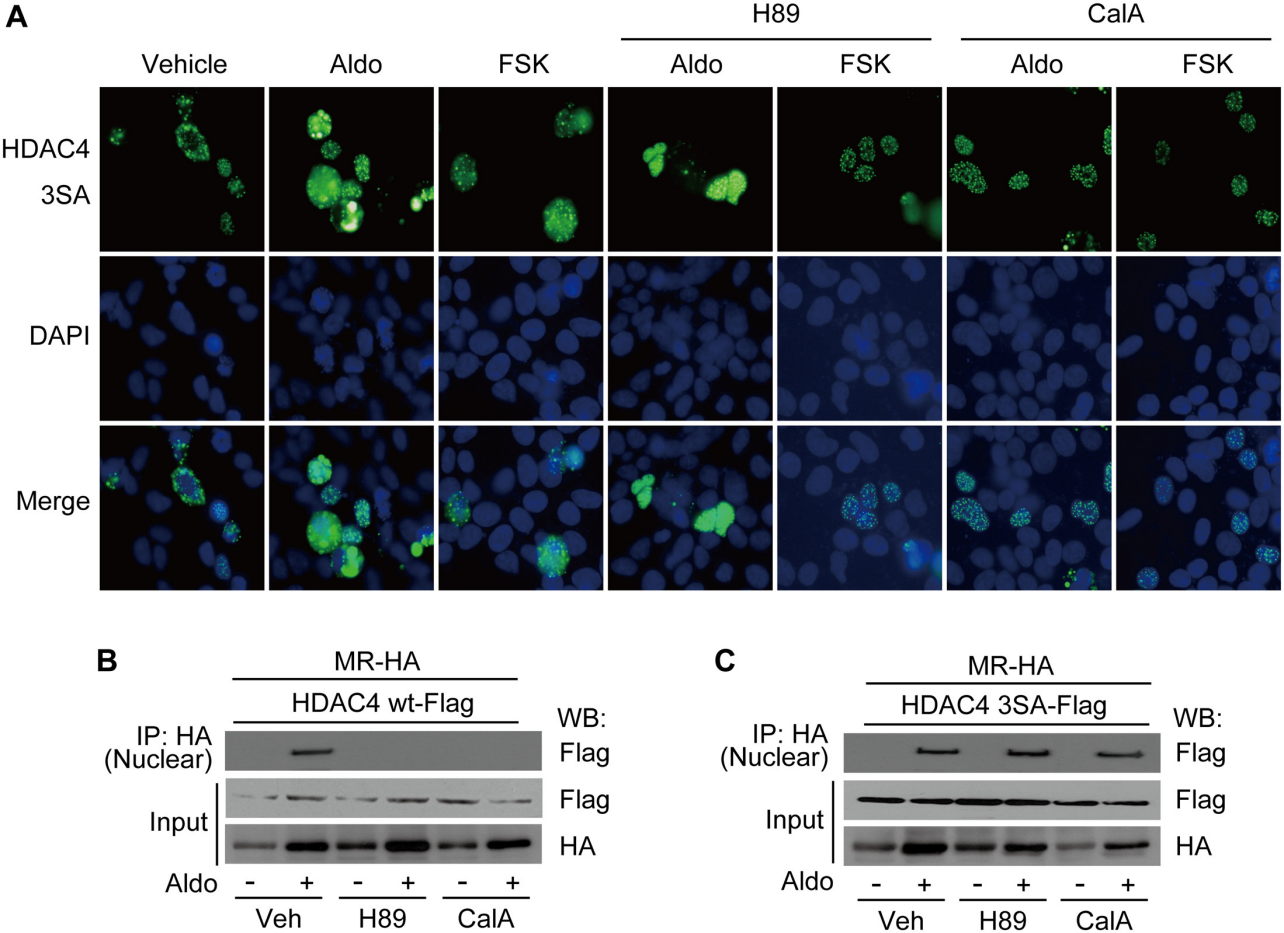
Location of mutant HDAC4 is not affected by H89 or calyculin A. A, HEK293 cells were transfected with GFP-tagged-mutant HDAC4 plasmid. After 48 h of transfection, cells were stimulated with Aldo or FSK for 30 min with or without pretreatment with H89 or calyculin A for 6 h, and analyzed by fluorescence microscopy. HEK293 cells were co-transfected with HA-tagged MR and Flag-tagged wild-type HDAC4 (B) or mutant HDAC4 (C). The cells were stimulated with Aldo for 30 min with or without pretreatment with H89 or calyculin A, and then fractionated. The interaction between MR and HDAC4 in each fraction was analyzed by a co-immunoprecipitation assay.

We investigated the effect of H89 and calyculin A on the transcriptional activity of MR, because H89 and calyculin A showed inhibitory effects on HDAC4 translocation (Fig 5). In addition, HDAC4 knockdown decreased the transcriptional activity of MR (Fig 3). Expression of *GILZ* (Fig 6A) and *SGK-1* (Fig 6B) induced by Aldo was significantly inhibited by pretreatment of cells with H89 (GILZ: ~74%; SGK-1: ~48%) or calyculin A (GILZ: ~68%; SGK-1: ~39%). Enrichment of MR and Pol II on the *GILZ* (Fig 6C) and *SGK-1* (Fig 6D) promoters induced by Aldo were also significantly reduced by H89 and calyculin A. The interaction between MR and HDAC3 induced by Aldo was also inhibited by H89 and calyculin A treatment (Fig 6E). However, MR translocation into the nucleus was not affected by H89 or calyculin A (Fig 6E). Since H89 and calyculin A treatments decreased the interaction between MR and HDAC3, we investigated the effect of H89 and calyculin A on MR acetylation. H89 and calyculin A resulted in increased acetylation of the MR when HEK293 cells were stimulated with Aldo (Fig 6F). Previous studies showed that dephosphorylation by PP2A induced the translocation of HDAC4 from the cytosol into the nucleus [24, 25]. The effect of HDAC4 phosphorylation on its localization was investigated using phosphomutant HDAC4 (S246A, S467A, S632A). Strikingly, phosphomutant HDAC4 was nuclear even without Aldo stimulation, and H89 and calyculin A had no effect on the nuclear localization of phosphomutant HDAC4 (Fig 7A). Aldo induced an interaction between MR and wild-type HDAC4 in the nucleus of HEK293 cells, which was inhibited by H89 and calyculin A (Fig 7B). In contrast, although phosphomutant HDAC4 interacted with MR in the nucleus of cells treated with Aldo, this association was not affected by H89 and calyculin A (Fig 7C).

## Discussion

We report here that the histone deacetylase 3 and 4 complex stimulates the transcriptional activity of the mineralocorticoid receptor. Aldo induces the translocation of HDAC4 from the cytosol to the nucleus. A non-genomic effect of MR activates PKA and PP1/2, which results in the nuclear import of HDAC4. Nuclear HDAC4 mediates the interaction between MR and HDAC3, which catalyzes MR deacetylation and enhances its transcriptional activity in our *in vitro* study.

Our previous report demonstrated that HDAC3 acts as a coactivator of MR. HDAC3 inhibition increases MR acetylation, which reduces its transcriptional activity and prevents the development of hypertension in deoxycorticosterone acetate-induced hypertensive rats [1]. However, we could not determine whether MR interacts directly or indirectly with HDAC3 in the previous study. Our new data show that HDAC4 simultaneously interacts with both MR and HDAC3 after treatment with Aldo. HDAC4 knockdown reduced the interaction between MR and HDAC3. In addition, HDAC4 knockdown increased the acetylation of MR. These results are very similar to those showing the regulation of FOXO transcriptional activation, a transcription factor that regulates cell metabolism [26]. HDAC4 recruits FOXO to HDAC3, deacetylating FOXO and activating FOXO target gene expression such as glucose-6-phosphate dehydrogenase [17]. The transcriptional activity of MR is significantly reduced by HDAC inhibitors, which increase acetylation in the MR hinge region. HDAC3 has been shown to play a central role in MR deacetylation, which increases the recruitment of MR and RNA polymerase II to the promoters of MR target genes and enhances their expression [1]. Increased acetylation of MR by HDAC4 knockdown resulted in a significant decrease in the Aldo-induced enrichment of MR and Pol II to the *GILZ* and *SGK-1* promoters, which is coincident with the expression of *GILZ* and *SGK-1*, respectively. However, the enzymatic activity of HDAC4 showed little effect on the transcriptional activity of MR since the Aldo-induced expression of MR target genes including *GILZ* and *SGK-1* was significantly decreased by MS-275, a specific inhibitor of HDAC1 and HDAC3, whereas MC1568, a specific inhibitor of HDAC4 and HDAC6, showed little effect on the expression of target genes. Moreover, MS-275 treatment reduced the recruitment of MR and Pol II to the promoters of MR target genes, whereas MC1568 showed little effect on their recruitment. These results may be related to MR acetylation, since MS-275 but not MC1568 increased MR acetylation. Interestingly, inhibition of HDAC4 activity did not block the interaction between MR and HDAC3. Taken together, these data indicate that HDAC4 has an important role as a scaffold between MR and HDAC3, a function independent of its deacetylase activity. HDAC5 also interacted with MR after Aldo stimulation. However, HDAC5 knockdown showed little effect on the expression of MR target genes, interaction between MR and HDAC3, and acetylation level of MR (S2 Fig). Several studies have revealed that class IIa HDACs (HDAC4, 5, 7, and 9) are catalytically inactive because of critical amino acid substitutions within their active sites [4, 5]. Although some studies have reported enzymatic activity of class IIa HDACs, this could be attributed to the association and recruitment of active HDAC3 and its co-regulators, such as nuclear receptor co-repressor1/silencing mediator of retinoic acid and thyroid hormone receptor [7].

**Fig 8:**
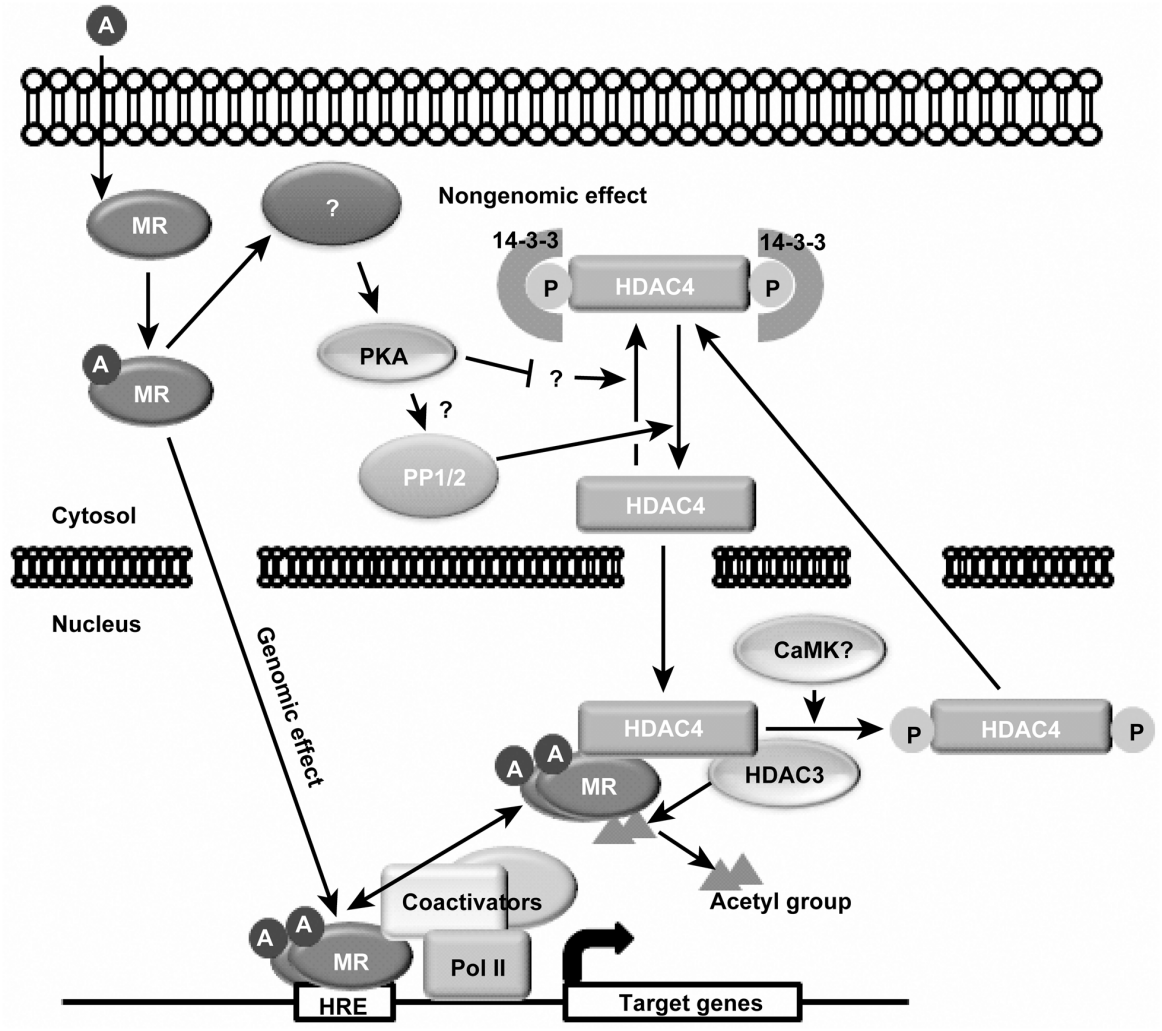
Summary of the present study. Ligand-bound MR translocates from the cytosol to the nucleus where it acts as a transcription factor (genomic effect of the MR). HDAC3 deacetylates MR, which increases MR DNA binding affinity. Interaction between MR and HDAC3 is mediated by HDAC4, which is imported to the nucleus through PKA and PP1 and 2 pathways (non-genomic effect of MR).

Class II HDACs are phosphorylated by several protein kinases including SIK, calcium/calmodulin-dependent protein kinase, and protein kinase D [8]. Phosphorylated class II HDACs interact with 14-3-3 proteins, which results in their sequestration in the cytoplasm. Loss of this interaction allows class II HDACs to translocate from the cytosol into the nucleus (11). Several studies have revealed that PKA and PP2A are involved in the nuclear translocation of class II HDACs [9, 13, 22, 25]. It is well defined that PKA indirectly causes the dephosphorylation of HDAC4 by inhibiting the activity of SIK2 and SIK3, which are responsible for phosphorylating serine 246, a binding motif for 14-3-3 proteins [13, 17]. Recent papers revealed that PKA also induces the dephosphorylation of serine 266, located in the nuclear localization signal of HDAC4, through unknown pathways [12]. Taken together, these studies indicate that PKA stimulates the nuclear translocation of class II HDACs. Paroni and colleagues showed that HDAC4 interacts with the A, B, and C subunits of PP2A, the inhibition of which by okadaic acid increases the phosphorylation of HDAC4 [9]. Additionally, Ling and colleagues found that casein kinase 2-interacting protein-1 regulates the interaction between HDAC4 and PP2A [25]. In accordance with previous reports, the PKA inhibitor H89 and the PP1 and PP2 inhibitor calyculin A blocked FSK- and Aldo-induced translocation of HDAC4 from the cytosol into the nucleus. Furthermore, treatment with H89 and calyculin A, which inhibit the translocation of HDAC4 into the nucleus, resulted in a significantly reduced interaction between wild-type HDAC4 and MR. However, phosphomutant HDAC4 (S246, S467A, S632A) was predominantly localized in the nucleus despite pretreatment with H89 and calyculin A and interacted with MR when cells were stimulated by Aldo. Inhibition of PKA and PP2A reduced the expression of MR target genes and recruitment of MR and Pol II to the promoters of MR target genes. FSK-induced nuclear translocation of HDAC4 was significantly inhibited by calyculin A. We surmise that the effect of PKA on HDAC4 is potentiated by those of PP1 and PP2 because FSK-induced HDAC4 translocation was blocked by calyculin A. Taken together, these results indicate that dephosphorylation of HDAC4 by PKA and/or PP2A is crucial for the interaction with MR, which is a pivotal cascade for increasing the transcriptional activity of MR. The results of the present study are summarized in Fig 8.

Aldo is one of the final effectors of the renin/angiotensin/aldosterone system. MR regulates salt balance and water homeostasis through classical pathways that induce specific ion channels and transporters (*e*.*g*., ENaC and ATP1a1), as well as specific mediators including GILZ and SGK-1 [27]. In addition, it is well known that non-classical pathways of MR exert several physiological and pathological responses [28]. One of the non-genomic effects of Aldo is that it acts as a physiological agonist that induces rapid increases in cAMP levels in cells such as vascular smooth muscle [29] and inner medullary collecting duct [30]. Therefore, we investigated whether Aldo induces PKA activity through a non-genomic effect using FRET. Aldo-induced PKA activity was found to proceed in a spatiotemporal manner (Fig 4A). PKA activity increased in the cytoplasm and reached a plateau within fifteen minutes after Aldo treatment (Fig 4B). This time course is well matched with the Aldo-induced nuclear translocation of the MR shown in a previous study [1].

Thus far, several broad-spectrum inhibitors of class I and class II HDACs have been developed and used as anti-cancer therapeutic drugs [31, 32]. Emerging evidence shows that these broad-spectrum inhibitors also show efficacy in treating a variety of diseases such as inflammatory, cardiovascular, and metabolic diseases [33]. However, the mechanisms of their therapeutic activities remain unclear. The development of selective inhibitors for each HDAC isoform is still in its infancy. However, these inhibitors likely have the potential to reveal the individual roles of these HDACs in the etiology of several diseases and may be used as more selective non-toxic therapeutic agents. Thus, the development of selective inhibitors should be accompanied by investigations that uncover the specific role of each HDAC isoform and its regulatory mechanism.

In conclusion, the present study demonstrates that Aldo induces the translocation of HDAC4 into the nucleus, where it acts as a scaffold between the MR and HDAC3 in our *in vitro* study. The translocation of HDAC4 was regulated at least by PKA and PP1 and PP2, which are activated by a non-genomic effect of MR. Nuclear HDAC4 facilitates the interaction between MR and HDAC3, an important enzyme that catalyzes the deacetylation of the MR.

## Supporting Information

S1 Fig Enrichment of MR and RNA Pol II on GRE of target gene promoters. Enrichment of MR and Rol II on *GILZ* (A) and *SGK-1* (B) promoters were analyzed by ChIP assay. Soluble chromatin was precipitated with anti-IgG, anti-MR, or anti-Pol II. Enrichment of MR and Pol II on HRE fragment and about 2 Kb upstream from HRE fragment (as a negative control) was determined by qPCR. Graph shows the means ± SE of three independent experiments (*p < 0.05 vs. vehicle).
(TIF)

S2 Fig HDAC5 had little effect on transcriptional activity of MR. A, Gilz expression was analyzed by qRT-PCR after knockdown of HDAC5. HDAC5 knockdown has little effect on expression of Gilz. Graph shows mean±SE of three independent experiments. B, Interaction between MR and HDAC3 was analyzed by coIP. HDAC5 knockdown shows little effect on interaction between MR and HDAC3. C, MR acetylation level was detected by anti-acetyl lysine antibody after immunoprecipitation with HA IP. HDAC5 knockdown has little effect on acetylation level of MR.
(TIF)

S1 Table Primer sequences for qRT-PCR and ChIP assay. (DOCX)
