# Supplementary figures and images for: Histone Deacetylase 3 and 4 Complex Stimulates the Transcriptional Activity of the Mineralocorticoid Receptor
Source: PLoS One. 2015 Aug 25;10(8):e0136801. doi: 10.1371/journal.pone.0136801 (PMC4549324; doi:10.1371/journal.pone.0136801)

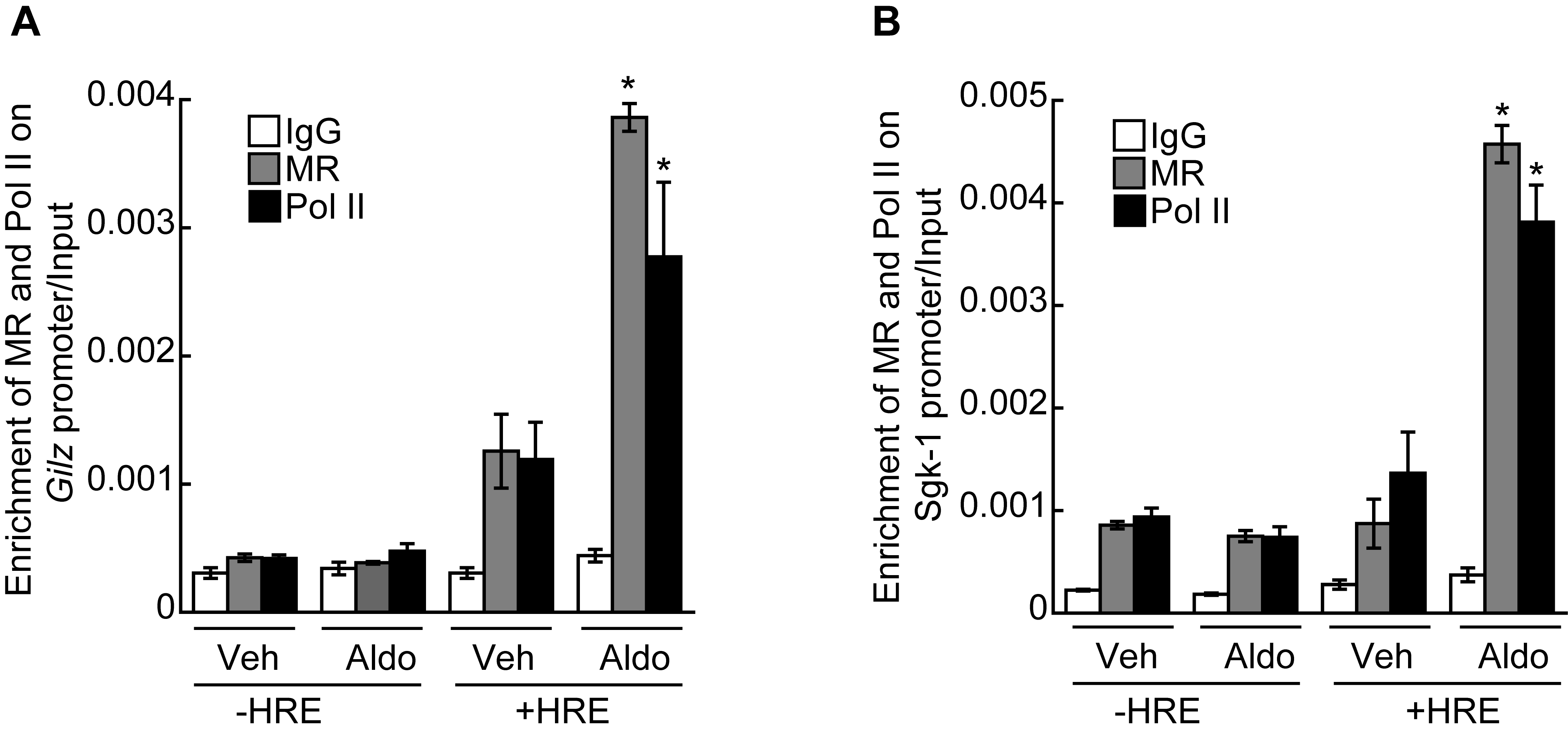

Supplement: S1 Fig — Enrichment of MR and Rol II on GILZ (A) and SGK-1 (B) promoters were analyzed by ChIP assay. Soluble chromatin was precipitated with anti-IgG, anti-MR, or anti-Pol II. Enrichment of MR and Pol II on HRE fragment and about 2 Kb upstream from HRE fragment (as a negative control) was determined by qPCR. Graph shows the means ± SE of three independent experiments (*p < 0.05 vs. vehicle). (TIF) [file pone.0136801.s001.tif]

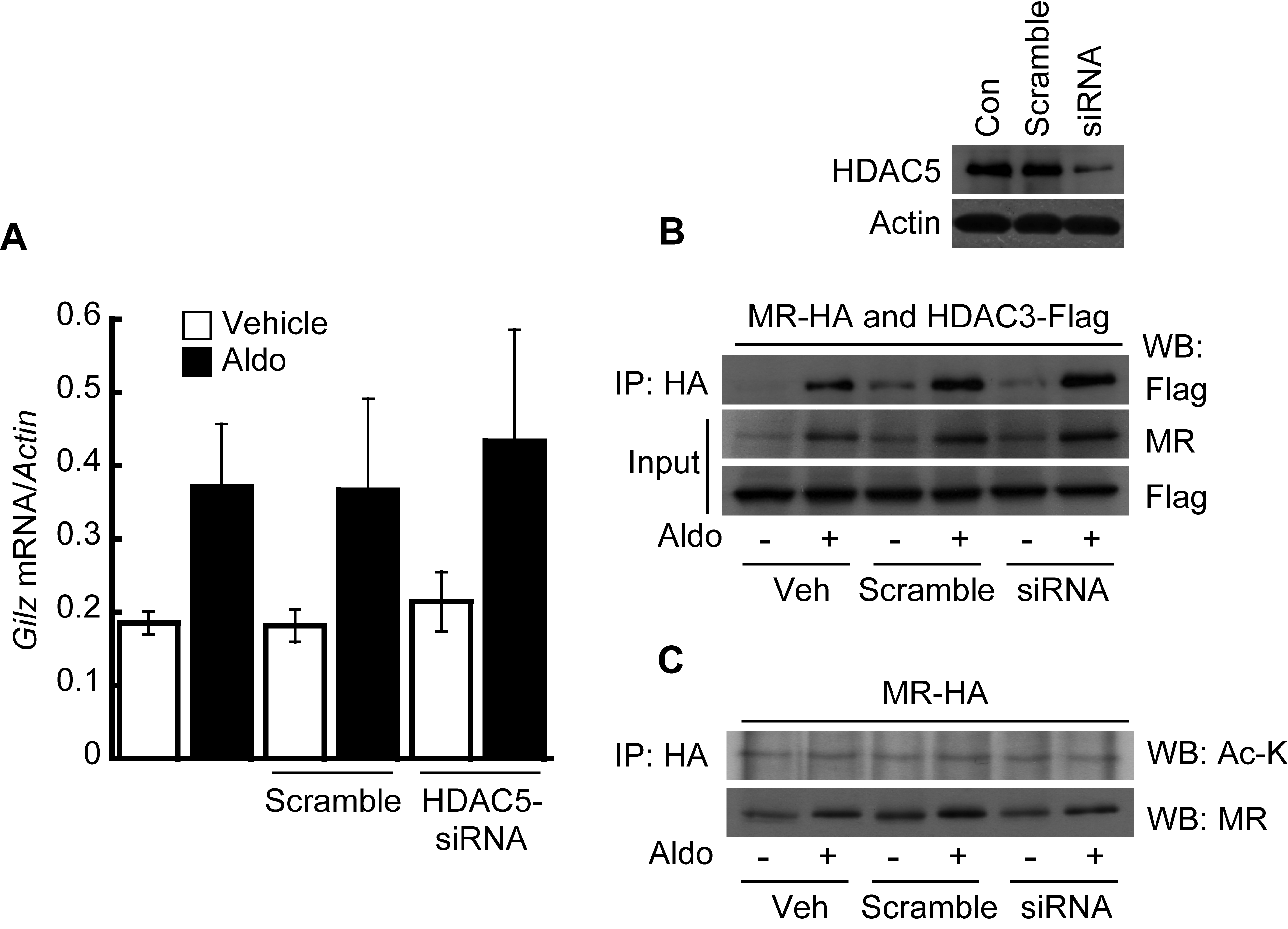

Supplement: S2 Fig — A, Gilz expression was analyzed by qRT-PCR after knockdown of HDAC5. HDAC5 knockdown has little effect on expression of Gilz. Graph shows mean±SE of three independent experiments. B, Interaction between MR and HDAC3 was analyzed by coIP. HDAC5 knockdown shows little effect on interaction between MR and HDAC3. C, MR acetylation level was detected by anti-acetyl lysine antibody after immunoprecipitation with HA IP. HDAC5 knockdown has little effect on acetylation level of MR. (TIF) [file pone.0136801.s002.tif]
