# Supplementary material for: Histone Deacetylase 3 and 4 Complex Stimulates the Transcriptional Activity of the Mineralocorticoid Receptor
Source: PLoS One. 2015 Aug 25;10(8):e0136801. doi: 10.1371/journal.pone.0136801 (PMC4549324; doi:10.1371/journal.pone.0136801)
Supplement: S1 Table — (DOCX) [file pone.0136801.s003.docx]

| Gene  (Accession No.) | Primer sequence  (5’ to 3’) | | Tm | | Amplicon Size (bp) | |  |
| --- | --- | --- | --- | --- | --- | --- | --- |
| qRT-PCR | | | | | | |  |
| GILZ  (NM_198057) | F:  R: | TGACACCAGTTTGCTCCAGA  GGCTGACTTGGCTCAATCTC | | 60 | | 190 | |
| SGK1  (NM_001143676) | F:  R: | GCAGAAGGACAGGACAAAGC  CAGGCTCTTCGGTAAACTCG | | 60 | | 185 | |
| ChIP assay | | | | | | |  |
| GILZ (+HRE) | F:  R: | ACCAGCTTCCATGTCTCACA  ATGTCTTGGGTGTGGAGGAG | | 60 | | 183 | |
| GILZ (-HRE) | F:  R: | GAGTTTGAGACCAGCCTGGG ATTACAGGAGCCACCAAGCC | | 60 | | 173 | |
| SGK-1 (+HRE) | F:  R: | CCTTCCTCATCATTAAATGGATTC  CCCCTCCCCTACTTGTCATT | | 60 | | 181 | |
| SGK-1 (-HRE) | F:  R: | GGAGTGAGGTCGTTTGCTCT GGCCATGCTAGCACTCAACA | | 60 | | 158 | |

**S1 Table**. Primer sequences for qRT-PCR and ChIP assay
